# The development and validation of the multidimensional fear-of-injection scale

**DOI:** 10.1080/21642850.2022.2116328

**Published:** 2022-08-29

**Authors:** Suzuka Hako, Kohei Kambara, Akiko Ogata

**Affiliations:** a Graduate School of Humanities and Social Sciences, Hiroshima University, Higashi-Hiroshima City, Hiroshima, Japan; b Faculty of Psychology, Doshisha University, Kyotanabe-shi, Kyoto-fu, Japan

**Keywords:** Fear of needles, direct fear, indirect fear, physiological response, avoidance behavior

## Abstract

**Background:**

Adults and children alike can feel anxious and afraid of needles. As a result, some people avoid necessary medical injections, leading to health problems. Assessing the fear of injections should consider fear factors, avoidance behaviors, and physical symptoms. We have developed a single scale that measures each of these variables. Conventional fear-of-injection scales do not simultaneously measure the aforementioned components, and thus may not adequately capture the fear of injections. Furthermore, no scale has been developed in Japan to measure the fear of injections.

**Method:**

A multidimensional fear-of-injection scale was developed in Study 1. The participants, 419 university students, were administered a questionnaire to check the reliability and validity of the scale. In Study 2, to establish the cut-off value of the scale, we conducted a questionnaire and analyzed the data of 771 university students. The outcome is the multidimensional fear-of-injection scale.

**Results:**

The results from factor analysis showed that this scale has a four-factor structure (direct fear, indirect fear, physiological response, and avoidance behaviors). The results of the receiver operating characteristic analysis showed that a cut-off value of 35 points identifies people with a subjective fear of injections.

**Conclusion:**

The multidimensional fear-of-injection scale is a comprehensive measure of the fear of injections and serves as an effective indicator for intervention and screening. Additionally, it provides a quantitative assessment of the fear of injection in Japan.

## Introduction

Needle procedures (e.g. vaccinations and venipunctures) are common methods used in clinical practice that most people experience. These medical procedures, often painful and stressful, are a well-known and essential component of medical treatment. While many children and adults can manage their apprehension and successfully undergo needle procedures, some feel great anxiety and fear when facing needles (McMurtry et al., 2016), and subsequently tend to avoid these procedures. A major consequence of the fear of needles is the tendency to evade medical procedures, such as injections. Accordingly, one may not receive appropriate medical treatment, thereby exacerbating illness, and in worst-case scenarios, leading to death (Olatunji et al., 2010). People who fear needles are twice as likely to avoid vaccination, including the COVID-19 vaccination, than those who do not (Freeman et al., 2021). It has been suggested that the fear of needles can affect the management of chronic diseases (e.g. insulin injections for diabetes) (McMurtry et al., 2015). Expressly, the lack of preventive care and disease management at the proper time may increase the risk of disease and a worsening health status, resulting in higher health care costs (McMurtry et al., 2015). Furthermore, a fear of needles narrows one’s range of career choices in the medical field (Milovanović et al., 2017).

Based on the diagnostic criteria of the Diagnostic and Statistical Manual of Mental Disorders, Fifth Edition (DSM-5) and the mechanism of specific phobias, there are three major constituents of the fear of needles. The first constituent is fear; according to Rachman (1977), fear is divided into two types: direct fear and indirect fear. While direct fear is the fear acquired by direct stimuli, such as direct conditioning, indirect fear is the fear acquired by indirect stimuli, such as observational learning or instruction from others. DSM-5 states that the development of phobia symptoms may occur after witnessing others encounter traumatic events and after the transmission of information (American Psychiatric Association [APA], 2013). Furthermore, according to Nir, Paz, Sabo, and Potasman (2003), observing others receiving injections, the size of the injections, and the pain experienced can be predictors of the fear of needles.

The second constituent is a physiological response to fear. DSM-5 states that vasovagal fainting is a characteristic response to the fear of needles. This is caused by an initial brief acceleration of heart rate and elevation of blood pressure, followed by a deceleration of heart rate and a drop in blood pressure (APA, 2013). Since fainting is a predictor of the fear of needles (Nir et al., 2003), physical symptoms as inner responses to fear are considered one of the constituents of the fear of needles.

The third constituent is avoidance as an external reaction to fear. According to Mowrer’s two-factor theory (Mowrer, 1951), fear is maintained through avoidance behavior. In addition, avoidance is considered an important factor because it is included in the specific phobia diagnostic criteria. Since medical avoidance is at the core of the fear of needles (APA, 2013), considering the avoidance of injection-related stimuli can help capture the severity of the fear of needles.

People with a high fear of injections tend to avoid medical situations and are less likely to notice problems due to their fear. As a chronic fear of injections can cause various issues, early screening is important. Globally, different fear-of-injection scales are used for this purpose, for example, the blood/injection fear scale (B/IF) (Kose & Mandiracioglu, 2007) and the injection phobia scale-anxiety (IPS-Anx) (Öst, Hellström, & Kåver, 1992). The blood-injection symptom scale (BISS) (Page, Bennett, Carter, Smith, & Woodmore, 1997) measures the degree of symptoms, and the phobia origins questionnaire (POQ) (Ollendick & King, 1991) measures the origin of fear. The IPS-Anx is a specialized scale that measures fear and consists of two factors: contact fear (e.g. injecting into the upper arm) and distal fear (e.g. seeing the picture of a person receiving the injection). The POQ is a questionnaire that investigates the acquisition of fear and consists of a specialized questionnaire for measuring Rachman’s three pathways. The BISS is a specialized scale for measuring the symptoms of the fear of injections.

These questionnaires are specialized scales that measure one aspect of the fear of injections. However, as mentioned earlier, the fear of needles is composed of various elements. Therefore, it is necessary to develop an optimum fear-of-injection scale for screening or to use as an effective indicator. Since the conventional fear-of-injection scales do not measure the four constituent elements of the fear of needles simultaneously, there is a possibility that the fear, in its entirety, is not captured. Therefore, the purpose of this study is to develop a single scale that includes a comprehensive set of components of the fear of needles. Specifically, in Study 1, we aim to develop the scale and test its reliability and validity. In Study 2, we aim to examine the cut-off values of the scale developed in Study 1.

## Study 1

### Method

#### Development of a new fear-of-injection scale

Prior to developing the scale, the permission was obtained from the scale’s original authors to translate the existing scale from English to Japanese.

Based on the existing fear-of-injection scales (e.g. Kose & Mandiracioglu, 2007; Öst et al., 1992; Page et al., 1997), with the help of clinical psychology graduate students and qualified faculty members, we gathered the relevant items. Twenty items were extracted by considering four factors (‘direct fear,’ ‘indirect fear,’ ‘physiological response,’ and ‘avoidance’). The items were selected based on their content and high factor loadings. After item selection, three graduate students majoring in clinical psychology confirmed and corrected the contents and developed a fear-of-injection scale. Table 1 shows the questions used to develop the fear-of-injection scale, the factors assumed, and reference questions. We named this new scale the Multidimensional Fear-of-Injection Scale (MFIS).

**Table 1.** Items in the developed fear-of-injection scale, factors assumed, and reference questions.

Assumed factors	Items	Reference items
Direct fear	Needle size frightens me	B/IF_7
	Having a shot in the upper arm	IPS-Anx_2
	I’m afraid of pain due to receiving injection	B/IF_1
	When I approach a hospital, I feel uneasy	DFS_9
	Sensing the smell of a hospital	DFS_12, IPS-Anx_4
Indirect fear	Watching a person in a nurse uniform	IPS-Anx_14
	Listening to someone talking about injections	IPS-Anx_10
	Watching a film about a person getting a shot	IPS-Anx_12
	Looking at a picture with a syringe and needle	IPS-Anx_3
	Looking at a picture of a person getting a shot	IPS-Anx_9
Physiological response	Did you faint?	BISS_8
	Did you feel nauseous?	DFS_6, BISS_12
	Were you dizzy or lightheaded?	BISS_5
	I have had rapid breathing	DFS_4
	Did you sweat?	DFS_5, BISS_14
	Did your heart pound?	DFS_7, BISS_10
Avoidance	I avoid receiving injections	B/IF_6
	I avoid to watch the nurse prepare the syringe	B/IF_3
	I have postponed an appointment in which an injection procedure was scheduled	DFS_1
	I have failed to attend an appointment in which an injection procedure was scheduled without giving notice	DFS_2

Note*.* B/IF = the Blood/Injection Fear Scale, IPS-Anx = the Injection Phobia Scale-Anxiety, DFS = Dental Fear Survey, BISS = The Blood-Injection Symptom Scale.

#### References for item collection

The B/IF (Kose & Mandiracioglu, 2007) consists of 20 items on a five-point Likert scale. The scale ranges from 1 (Maximum Fear) to 5 (No Fear). The scores range from 20 to 100 points. The lower the score, the higher the stress level. It also consists of two factors: injection concern and blood concern. Items 1, 3, 6, and 7 were used for scaling.

The BISS (Page et al., 1997) consists of 17 items in which individuals rate their degree of symptoms, such as fear and fainting. Responses were scored on a two-point scale (Yes/No). The scoreable range is from 0 to 17 points, and items 5, 8, 10, 12, and 14 were used for scaling.

The IPS-Anx (Öst et al., 1992) measures individuals’ degree of fear and anxiety if they are to experience various injection procedures. It consists of two factors: distal fear and contact fear. This scale comprises 18 items rated on a five-point Likert scale. The scale ranges from 0 (Absolutely Uneasy) to 4 (Very Difficult). Items 2, 3, 12, and 14 were used for scaling. The factor structure, reliability, and validity were confirmed by Olatunji et al. (2010).

The dental fear survey (DFS) (Sano, Tanabe, & Noda, 2001) consists of three factors: avoidance of dentistry, physiological response, and fear of the stimulus. Since this scale was originally designed to assess the fear surrounding dental procedures, the wording was revised in items 1, 2, 4, 5, 6, 7, 9, and 12 to reflect relevance for an injection procedure.

#### Participants

In this study, we sent questionnaires to 423 university students; four were excluded from the analysis because they stopped responding. Therefore, the number of effective respondents was 419. Participants’ ages ranged from 18 to 25 years (*M_age_* = 20.00; SD = 1.39; 200 males, 217 females, and two with gender not specified).

#### Measurement items

The MFIS (Table 1), DFS, and state-trait anxiety inventory (STAI-T) were used in this study. The MFIS was recorded on a five-point Likert scale (1 = Absolutely Not Applicable, 5 = Very Applicable). The scale consisted of 20 items. The higher the score, the greater the degree of the fear of injections.

The DFS (Sano et al., 2001) found that individuals with a fear of needles experienced strong symptoms at the time of dental treatment and a high possibility of avoiding subsequent dental treatment (McMurtry et al., 2015). Dental fear is an additional predictor of the fear of needles (Milovanović et al., 2017). Therefore, it was used to investigate the validity of the fear-of-injection scale. The DFS consists of 20 items and measures fear of dental on a five-point Likert scale (1 = No, 5  = Yes).

The STAI-T (Shimizu & Imae, 1981) found that anxiety is a predictor of the fear of needles (Milovanović et al., 2017), and that individuals with anxiety are anticipated to be fearful of injections (McMurtry et al., 2015). Therefore, it was also used to investigate the validity of the fear-of-injection scale. The STAI-T consists of 20 items and measures trait anxiety on a four-point Likert scale (1 = Not At All, 4 = Absolutely So).

#### Procedure

We conducted surveys by distributing questionnaires during university lectures and recruited participants through personal connections as well. When surveying the lecture, we first administered an oral explanation of the content of the survey. In the case of a homework survey, we informed participants that the survey would be collected after one week, and in the case of a simultaneous survey, the participants were informed that they would be asked to answer questions on the spot. While recruiting through personal connections, we explained our study requirements orally or in writing and handed the questionnaire to the participants. Following this, each participant answered the questionnaire, which was then collected.

#### Ethical considerations

This study was approved by the Ethics Review Committee, Graduate School of Education, Hiroshima University. The purpose of the research and ethical and social considerations were specified on the front cover of the questionnaire. We communicated the research’s content orally or in writing to the target participants when distributing the questionnaire. The survey was conducted anonymously; participation was arbitrary. We informed participants that if they refused to complete the questionnaire, it would not be to their detriment. Participants were considered to have agreed to participate in the study upon completing the questionnaire.

#### Data analysis

Exploratory factor analysis (EFA) (maximum likelihood method, determination of the number of factors by scree plot, and Promax rotation) was used to examine the factor structure. Items with factor loadings less than 0.40 were deleted. To verify reliability, Cronbach's α coefficient was used. To investigate the relevance of the criterion, we examined the relationship between the Japanese version of the STAI-T and the DFS. Finally, confirmatory factor analysis was performed based on the constitutive concept obtained by EFA. The goodness of fit index (GFI), adjusted GFI (AGFI), comparative fit index (CFI), and root mean square error of approximation (RMSEA) were used as indicators of model fitness. We used HADon16_031 (Shimizu, 2016) for statistical analysis.

### Results

To confirm the structure of the developed fear-of-injection scale, EFA was performed. There were four items (item 2, 8, 14, 18) whose factor loadings did not reach 0.40; these were discarded and EFA was performed again. As a result, 16 out of 20 items were adopted. The number of factors was determined by the scree plot. The factor loadings, correlations between factors, and reliability factors are listed in Table 2. The model fit was GFI =  .873, AGFI = .824, CFI = .890, and RMSEA = .096. The scales were labeled as follows: direct fear (5 items), indirect fear (4 items), physiological response (4 items), and avoidance (2 items). The mean total score on the MFIS was 31.71, with a standard deviation of 11.54 for 419 participants. The scores ranged from to 16–80. Cronbach’s alpha was 0.79, indicating high internal consistency. The alpha for each factor was .842, .876, .822 and .782, respectively. Convergent validity was assessed using Pearson’s correlations between the MFIS, DFS, and STAI-T. The MFIS score indicated a statistically significant positive correlation with the DFS (*r* = .53; *p *< .01) and STAI-T (*r* = .25; *p *< .01). The mean, standard deviation, score range, and Pearson’s correlations are listed in Table 3.

**Table 2.** Factor loadings, correlations between factors, and reliability factors.

Item		Direct fear	Indirect fear	Physiological response	Avoidance
5		**.844**	.013	-.048	.017
15		.**809**	.021	-.019	-.028
4		.**764**	.008	.009	-.039
1		.**673**	.017	-.071	.023
20		.**436**	.039	.220	.015
16		-.060	.**938**	.016	-.014
9		.063	.**734**	.068	.030
3		.189	.**717**	-.092	.004
7		.309	.**445**	.047	-.033
17		-.118	.066	.**891**	-.037
6		.012	-.056	.**874**	-.132
11		-.161	.078	.**573**	.130
12		.142	-.011	.**552**	.074
13		.276	-.100	.**491**	.110
10		-.008	.001	-.043	.**947**
19		-.007	-.003	.078	.**695**
2					
8					
14					
18					
	Interfactorial correlation	Direct fear	Indirect fear	Physiological response	Avoidance
	Direct fear		.684	.411	.217
	Indirect fear			.445	.281
	Physiological response				.383
	Avoidance				
	Cronbach’s alpha	.842	.876	.822	.782

**Table 3.** Correlation analysis.

	Range	Mean	SD	1		2		3		4		5		6		7
1.Fear of needles	16–80	31.71	11.54	1.000												
2.Direct fear	5–25	13.66	5.45	.879	**	1.000										
3.Indirect fear	4–20	8.39	4.41	.855	**	.681	**	1.000								
4.Physiological response	5–25	7.46	3.71	.704	**	.415	**	.425	**	1.000						
5.Avoidance	2–10	2.28	1.02	.375	**	.192	**	.231	**	.335	**	1.000				
6. Dental fear	20–100	35.78	16.46	.558	**	.496	**	.505	**	.361	**	.250	**	1.000		
7. STAI-T	20–80	48.77	9.99	.247	**	.222	**	.183	**	.220	**	.075		.246	**	1.000

Note*.*

**p < .01.

*p < .05.

^+^ *p *< .10.

SD = standard deviation

### Discussion

In this study, a new scale was developed to measure the fear of injections from multiple perspectives, and its factor structure, reliability, and validity were examined. Our findings were as follows:

- The MFIS consists of four factors: direct fear, indirect fear, physiological response, and avoidance.
- The MFIS had high reliability.
- The MFIS correlated with trait anxiety and dental fear.

The MFIS was constructed to measure a broad range of stimuli for symptoms of the fear of needles. Existing scales measuring fear-of-injection only consider a single aspect, such as fear stimuli, symptoms, or avoidance behaviors (Kose & Mandiracioglu, 2007; Öst et al., 1992; Page et al., 1997). In contrast, the MFIS can measure four aspects of the fear of needles, making it easier to perform a multifaceted assessment. As a result of the confirmatory factor analysis, the fidelity of the data was considered good. However, it is a limitation that the avoidance item consists of two items. Rating avoidance included specific actions, such as ‘not seeing a doctor/postponing an appointment.’ Therefore, it is likely that the items included measure more serious avoidance behaviors. Conversely, individuals with high values of avoidance were considered to have a serious level of feara of needles. However, since it is difficult to measure avoidance behavior in persons with moderate levels with this item, additional items would need to be added in the future.

Cronbach's α coefficients were determined to assess the scale’s reliability and the factor-by-factor reliability coefficients were above α = 0.75. The reliability coefficient of the overall scale was α = 0.89, which indicated secure reliability of the MFIS.

To further examine validity, we measured the Japanese version of the STAI-T and the DFS in relation to fear of injections and examined the correlations of each scale. There was a weak positive correlation between fear of injections and trait anxiety, and a moderate positive correlation between fear of injections and dental fear, indicating criterion-related validity. This suggests that the fear of needles is unlikely to be associated with general anxiety. The fear of needles and fear of dentistry are considered to be related but are different phenomena; the correlation with the fear of dental treatment, which is as invasive as an injection procedure, was moderate.

## Study 2

### Method

A total of 771 participants (384 women; M_age_ = 20.06 years; SD = 1.40) were included in the analysis, including the data from 361 university students from Study 1. This study measured two aspects: fear of injections and subjective fear of injections. To measure the fear of injections, the MFIS developed in Study 1 was used. It consists of 16 items on a five-point Likert scale. The scale ranges from 1 (No Fear) to 5 (Maximum Fear). The possible score ranges from 16 to 80 points. The higher the score, the higher the fear of injections. Regarding the subjective fear of injections, the participants were asked to answer whether they were afraid of injections at present, using the two-case method of ‘Yes’ or ‘No.’

As in Study 1, we recruited participants by distributing questionnaires during university lectures and through personal connections. ROC curve analysis was performed using EZR version 1.41 (Kanda, 2013) to investigate the accuracy of the MFIS discrimination of the fear of injections and optimal cut-off. In the ROC analysis, sensitivity (true positive rate—the rate at which a patient can be correctly judged positive in the presence of disease or disability) was set on the vertical axis and specificity (true negative rate—the rate at which a patient can be correctly judged negative in the absence of disease or disability) was set on the horizontal axis. The evolution of the two indicators was plotted while the cut-off value was continuously changed, and the ROC curve was drawn. The optimal cut-off value was the point at which the ROC curve was closest to the upper left corner of the graph.

### Results

**Figure 1.**
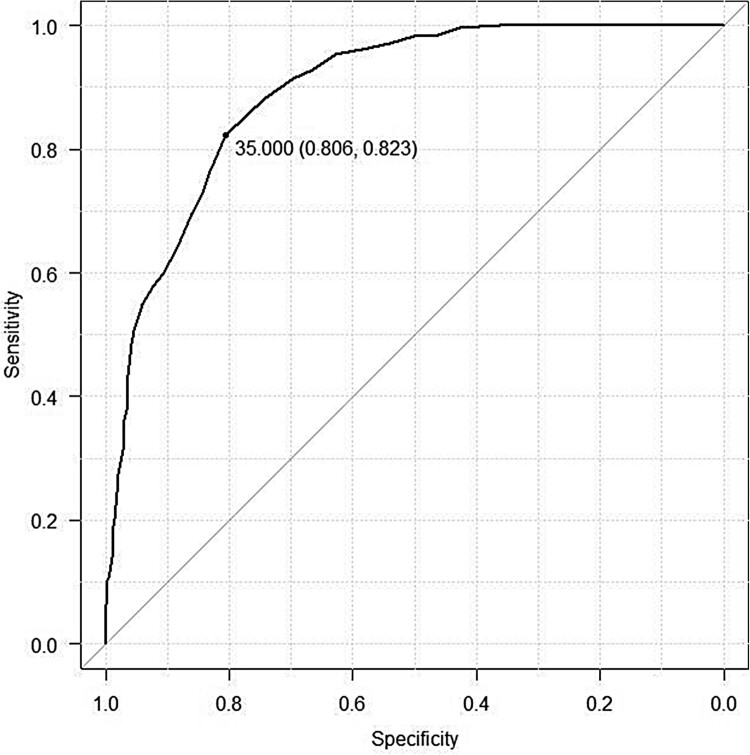
The plotted receiver operating characteristic curve.

The ROC curve was calculated using the current subjective fear of injection as the dependent variable and the MFIS score as the explanatory variable. The results revealed that the area under the curve (AUC), a measure of discrimination accuracy, was 0.89 (95% Cl: 0.87–0.92). Further, the sensitivity (true positive rate) and specificity (true negative rate) were high at 0.81 and 0.82, respectively, when the MFIS cut-off was set at 35 points. Therefore, 35 points was used as a cut-off to identify individuals with a fear of injections (Figure 1).

Per the results, 296 (38.34%) of the analyzed 771 participants were above the cut-off. Summary statistics for participants below the cut-off and those above the cut-off are presented in Table 4. There was a nearly two-fold difference in the fear-of-injection scores between those without a cut-off and those with a cut-off or higher. For each of the sub factors, direct fear was high below the cut-off, but a floor effect was found below the cut-off for indirect fear and physiological responses.

**Table 4.** Fear of injection below and above the cut-off and scores on each subscale.

	Range	Below cut-off		Above cut-off	
		Mean	SD	Mean	SD
Fear of needles	16—80	24.72	5.80	44.91	8.92
Direct fear	5—25	10.67	3.92	19.22	3.46
Indirect fear	4—20	6.05	2.33	12.92	3.95
Physiological response	5—25	5.90	1.59	10.07	4.73
Avoidance	2—10	2.10	0.53	2.63	1.52

Note. SD = standard deviation

### Discussion

The results of the ROC analysis indicated that the AUC of the MFIS was 0.89; therefore, the model was judged to have sufficient discriminatory power. We also examined the characteristics of the participants below and above the cut-off and found that 38% of them were above the cut-off; the prevalence of the fear of injections among people in their 20s in a meta-analysis by McLenon and Rogers (2019) ranged from 20 to 30%. The percentage of individuals beyond the cut-off of the MFIS was 38%, which is slightly higher than the prevalence.

Of the 297 participants beyond the cut-off, 99 (12.82%) answered ‘No’ to the question regarding a subjective fear of injections, indicating that they may meet the screening criteria even if they do not subjectively fear injections. In contrast, although the cut-off was not crossed, only 5.8% of the respondents answered ‘Yes’ to the question regarding a subjective fear of injections. Therefore, the cut-off values set in this study are generally appropriate for identifying people with a fear of injections. The MFIS is expected to be very useful in screening because it is less likely to overlook a fear of injections.

Additionally, floor effects were found for indirect fear and physiological responses in participants below the cut-off. As indirect fear is fear felt outside of the injection stimulus, those with a high tendency to fear injections beyond the cut-off may have a generalized fear of fear-arousing stimuli, such as images and videos. Regarding physiological responses, the high scores may be a result of excessive fear responses, such as sweating and elevated heart rate. In particular, the proportion of respondents who reported having experienced vasovagal syncope, a characteristic response to injection fear, was 8.75% for the cut-off and beyond, compared with 0.21% for the cut-off and below.

In terms of avoidance, only two items were included on the MFIS, which allows us to measure positive avoidance, such as canceling a scheduled injection appointment or postponing a scheduled injection appointment. For the avoidance items, there was an overall floor effect. Therefore, those with a rating of three or more on the avoidance items of the MFIS have a high probability of experiencing a fear of injections.

These results suggest that to identify individuals with a high tendency to fear injections, it is necessary to focus on indirect fear, physiological response scores, the overall fear-of-injection scores, and the tendency to respond to avoidance items rather than direct fear. In addition, the object and symptoms of fear differ from person to person. Therefore, the cut-offs may be useful in screening injection-phobic patients, and the total score may be useful in understanding individual conditions. Expressly, this scale can be used flexibly according to its purpose, such as screening for intervention studies or selecting intervention targets.

## Conclusion

The fear of needles is a condition, which, if left untreated, can cause serious health consequences. Therefore, establishing a short but reliable, valid, and sensitive assessment of the fear of injections is important for the purposes of both research and clinical evaluation (Olatunji et al., 2010). The MFIS developed in Study 1 can measure the four components of the fear of needles (direct fear, indirect fear, physiological response, and avoidance) on a single scale. In addition, the scale was found to have high reliability and validity. In Study 2, a cut-off value for an individual’s subjective fear of injections was established, allowing a high probability of identifying individuals with a fear of injections. Further, it is possible to describe the characteristics of an individual’s fear of injections from the distribution of scores on the subscales. Expressly, the MFIS is useful as an efficacy index and screening index.

However, the cut-off values established in this study are not intended to measure the presence or absence of a diagnosis. This is a limitation of this study. In the future, structured interviews should be conducted to establish cut-off values that meet diagnostic criteria.

In summary, the MFIS appears to be a valid and reliable instrument for measuring fear-of-injection symptoms. Unlike existing scales, the MFIS measures the fear of injections from multiple perspectives, and we hope that the MFIS will be adopted in clinical and research settings.

## Date availability statement

The data that support the findings of this study are available from the corresponding author, S.H., upon reasonable request.
